# Factors associated with self-rated health in people with late-stage parkinson’s and cognitive impairment

**DOI:** 10.1007/s11136-024-03703-2

**Published:** 2024-06-18

**Authors:** Jennifer S. Pigott, Megan Armstrong, Nathan Davies, Daniel Davis, Bastiaan R. Bloem, Stefan Lorenzl, Wassilios G. Meissner, Per Odin, Joaquim J. Ferreira, Richard Dodel, Anette Schrag

**Affiliations:** 1 Clinical Neurosciences, Queen Square Institute of Neurology, University College London, Royal Free Hospital, Rowland Hill Street, London, NW3 2PF UK; 2 Centre for Ageing Population Studies, Research Department of Primary Care and Population Health, University College London, London, UK; 3 Centre For Psychiatry and Mental Health, Queen Mary University of London, London, UK; 4 MRC Unit for Lifelong Health and Ageing, University College London, London, UK; 5 Donders Institute for Brain, Cognition and Behavior, Department of Neurology, Radboud University Nijmegen Medical Centre, Nijmegen, The Netherlands; 6 Institute for Palliative Care, Paracelsus Medical University, Salzburg, Austria; 7 Department of Palliative Medicine, University Hospital, LMU Munich, Munich, Germany; 8 Department of Neurology and Palliative Care, University Hospital Agatharied, Hausham, Germany; 9 Service de Neurologie des Maladies Neurodégénératives, IMNc, IMN, UMR 5293, CHU de Bordeaux, Univ. de Bordeaux, CNRS, 33000 Bordeaux, France; 10 Dept. Medicine, University of Otago, Christchurch, and New Zealand Brain Research Institute, Christchurch, New Zealand; 11 Division of Neurology, Department of Clinical Sciences Lund, Lund University, Skåne University Hospital, Lund, Sweden; 12 Faculdade de Medicina, Instituto de Medicina Molecular João Lobo Antunes, Universidade de Lisboa, Lisboa, Portugal; 13 Department of Geriatric Medicine, University Duisburg Essen, Essen, Germany

**Keywords:** Cognitive impairment, Parkinson’s disease, Parkinson’s disease dementia, Self-rated health.

## Abstract

**Purpose:**

To investigate the contributors to self-rated health in people with late-stage Parkinson’s disease (PD) and cognitive impairment.

**Methods:**

A secondary analysis of baseline data from the international Care of Late-Stage Parkinsonism (CLaSP) cohort study was conducted. Participants with PD and either dementia or mild cognitive impairment or MMSE < 24/30 in the absence of major depression were included if they had completed the EQ-5D-3L assessment (n = 277). Factors associated with self-rated health (EQ-5D-3L Index and Visual Analogue Scale) were investigated through multivariable linear regression.

**Results:**

More severe PD (motor and non-motor) was associated with worse self-rated health. The EQ-5D-3L dimensions of Mobility, Self-Care and Usual Activities were almost universally affected; the latter two particularly severely. Being unable to perform usual activities or having moderate to extreme anxiety or depression were significantly associated with EQ-5D-3L Visual Analogue Scale, suggesting these are particularly valued. Worse motor impairment and function and the non-motor symptom domains of mood, perception, sexual function, and miscellaneous (e.g., pain) were associated with worse self-rated health, whereas greater burden of gastrointestinal symptoms was associated with better self-rated health in multivariate analysis. Better self-rated health was associated with recent PD nurse consultation, and higher doses of dopaminergic medication.

**Conclusion:**

Improvement of activities of daily living, mood and anxiety should be prioritised in clinical practice, with consideration of perception and sexual function in this population. Recent nurse consultations and higher antiparkinsonian doses are associated with better self-rated health, suggesting there is no room for a therapeutic nihilism in this population of people within a complex phase of PD.

**Supplementary Information:**

The online version contains supplementary material available at 10.1007/s11136-024-03703-2.

## Plain English Summary

Parkinson’s is a complex and progressive health condition that causes a wide range of symptoms and significantly impacts daily life. ‘Cognitive’ problems include memory and thinking problems. These are common in Parkinson’s, particularly in later stages. However, research about the experience of people with Parkinson’s who have these symptoms is limited.

We sought to understand how people with Parkinson’s who have cognitive problems perceive their health, and what factors influence this.

We found that performing usual activities, low mood and anxiety are particularly important to people with this condition. A range of Parkinson’s-related problems, including low mood, were associated with how people perceived their health, as has been the case for other groups of people with Parkinson’s in past research. More severe movement problems, limitations in ability to perform activities, perception symptoms such as hallucinations, and sexual problems were important, and may be more specific to this group of people. We also found that people who had seen a Parkinson’s nurse within the last 3 months and people taking more Parkinson’s medication reported better health.

We suggest that the factors identified as important should be addressed as a priority by healthcare professionals. Our findings also show the importance of the Parkinson’s nurse role for people with Parkinson’s and cognitive problems, and in ensuring medication is reviewed.

## Background

Parkinson’s disease (PD) is a complex neurodegenerative condition with increasing prevalence [1] and progressive course, conferring a heterogeneous range of impairments. Advancing PD is characterised by increasing dependence on caregivers for activities of daily living (ADLs), owing to treatment-resistant motor or non-motor symptoms, including cognitive decline [2]. Cognitive symptoms are common in PD, increasing with PD duration and more severe PD [3, 4] and dementia is often considered a hallmark of advanced disease [5]. Cognitive impairment in PD is associated with increased dependence, higher caregiver burden and higher economic cost [4, 6–11]. Despite the high prevalence of cognitive impairment in advanced stages of PD, there is however relatively little information on patients’ health status from their point of view. Although many studies have evaluated factors associated with worse self-rated health in PD overall, often using PD-specific measures, most have been conducted in early to mid-stage disease (e.g. [12–15]), while people with dementia were often excluded [14, 16, 17] or not represented [13]. Several reviews have identified non-motor symptoms, in particular depression, and functional impairment or dependence as being key predictors of self-rated health in PD overall [18–21], and the impact of PD is more pronounced in advanced disease [20]. Impairment in cognitive function, particularly attention and executive function, has also been associated with poorer self-rated health even in early disease stages [22, 23]. Literature on this specific population is however sparse. A recent study from China investigated determinants of self-rated health according to cognitive status in PD, although in relatively early disease with relatively young participants (dementia group mean age 62.8 ± 6.93 years) [24]. Motor function was the strongest predictor of self-rated health for those with normal cognition, whereas depression was the strongest predictor for those with mild cognitive impairment or dementia. Bodily discomfort, cognition and mobility domains of the PDQ-39, a disease specific measure of self-rated health, were most affected for those with cognitive impairment.

To our knowledge, no studies have investigated determinants of self-rated health specifically for those with cognitive impairment in late-stage PD. Through greater understanding of factors that influence self-rated health, including healthcare and social care factors, interventions and services could be targeted to try to improve it.

## Objective

Our aim was to investigate the factors associated with self-rated health of people with PD and cognitive impairment.

## Methods

### Care of late-stage parkinsonism (CLaSP) study

This is a multi-centre, prospective cohort study of people with late-stage parkinsonism and caregivers (either individual patients or patient-caregiver dyads) over 18 months, conducted in six European countries: Germany, Portugal, Sweden, UK, France and the Netherlands. Late-stage parkinsonism was operationally defined by disease duration of seven years or more; and Hoehn and Yahr stage IV or V, or Schwab and England stage 50% or less in the “On”-state. Details of the study have been published previously [25]. Participant identification and recruitment was adapted to healthcare arrangements in each country to target this hard-to-reach group. Patients are seen to withdraw from specialized medical care once they reach advanced stages of PD [2], so a variety of recruitment methods were employed, with extensive efforts made to recruit patients beyond specialist settings to minimize selection bias. Primary and secondary care providers, community settings such as nursing homes, and patient organizations (self-help groups and advocates) were contacted. The study sites included neurology, care of the elderly, and palliative care settings. Data was collected by trained researchers, through face-to-face interviews with participants and their caregiver, with breaks and repeated visits provided as required to facilitate completion. Ethical approval was granted locally for each site, and participants provided consent.

### Participants

The CLaSP study included people with late-stage parkinsonism, as defined above, and excluded secondary parkinsonism or dementia with clear onset before motor symptoms. For the present analysis, baseline data for the subgroup of patients with Parkinson’s disease and cognitive impairment. Atypical and vascular Parkinsonism were excluded. Cognitive impairment was operationally defined as a pre-established diagnosis of dementia or Mild Cognitive Impairment, or a Mini Mental State Examination (MMSE) score < 24/30 in absence of major depression, as suggested by a score of 4 on the Unified Parkinson’s Disease Rating Scale (UPDRS) Part-I Question 3 (“Sustained depression with vegetative symptoms and suicidal thoughts or intent”). Participants who did not meet the disease duration of at least 7 years inclusion for the main CLaSP study could still be included in our analysis since the focus was cognitive impairment which can be marked even after relatively short disease duration [4] but is often considered an indicator of advanced Parkinson’s [26]. Analyses of variables only relevant to participants living in their own homes (professional and informal care provision) were applied to that subgroup.

### Assessments

A range of assessments were conducted during the CLaSP study [25]; those included in this secondary analysis are shown in Table 1. For the primary outcome of this analysis, self-rated health was assessed using the EuroQoL (EQ-5D-3L), since this was applied to all participants according to study protocol and has been validated for those with dementia in PD [27, 28]. It also allows comparison with other studies including non-PD populations. It is a two-part instrument: five dimensions are each assessed with one question with three possible response levels as shown in Table 1, plus participants are asked to indicate their overall perceived health status on a visual analogue scale (EQ VAS). The dimensions provide a description of self-rated health. An index is calculated to synthesise these dimension responses based on published population-specific value sets. For consistency and comparability we used the UK value sets [29, 30] for all participants as recommended by the EuroQoL group for international studies. Studies suggest that across Western European countries values are broadly similar [31, 32]. The range of possible scores for this calculated index is -0.594 to 1, with lower score indicating worse health status.

**Table 1:** Assessments

Domain	Assessment	Notes
Demographic	Gender	
	Age	
	Years of education	
	Marital Status	
Clinical – General	Diagnoses	Includes year of onset of Parkinsonism
	Medication	Levodopa Equivalent Daily Doses (LEDD) were calculated from reported medication [59]
Clinical Measures	Hoehn and Yahr Stage [36]	Higher stage indicates more advanced Parkinson’s
	UPDRS [60]	Part-I mentation, behaviour and mood; Part-II ADLs; Part-III motor examination; and Part-IV complications of therapy. Higher scores indicate more severe Parkinson’s
	MMSE [61]	Lower scores indicate greater cognitive impairment
	Non-Motor Symptom Scale (NMSS) [62]	Higher scores indicate greater burden of non-motor symptoms comprising severity and frequency
Care Measures	A resource utilisation questionnaire [63]	Completed by the person with PD and/or their caregiver. It covers multidisciplinary primary and secondary healthcare use as well as social care (professional and informal homecare and other services)
	Schwab & England scale [64]	To assess functional dependence. Range from 0–100% where higher scores indicate greater independence with daily functioning
	Zarit Burden Interview [65]	To assess impact on caregivers: higher scores indicate greater burden
Self-rated Health	EuroQoL (EQ-5D-3L) [27]	This assesses 5-dimensions each with 3 response levels: Mobility- *No problems in walking about; some problems walking about; confined to bed* Self-care- *No problems with self-care; some problems washing or dressing myself; unable to wash or dress myself* Usual Activities (e.g. work, study, housework, family or leisure activities): *no problems with performing my usual activities; some problems with performing my usual activities; unable to perform my usual activities* Pain/discomfort- *no pain or discomfort; moderate pain or discomfort; extreme pain or discomfort* Anxiety/depression- *not anxious or depressed; moderately anxious or depressed; extremely anxious or depressed* It also uses a Visual Analogue Scale (VAS) to assess overall perceived health status between the limits of 0 (worst imaginable health status) and 100 (best imaginable health status)

### Analysis

Statistical analysis was performed in Stata 17 [33]. Distributions were assessed visually. Descriptive statistics are presented as mean and standard deviation, and median and interquartile range (latter only for ordinal data), and numbers and percentages for categorical data, with numbers having completed each assessment presented. Correlation between the EQ-5D-3L Index and EQ VAS was assessed through Spearman rank correlation analysis. Relative contributions of the five dimensions of the EQ-5D-3L descriptive system to the EQ VAS were investigated by linear regression analysis with EQ VAS as the outcome. Missing data for the outcome variable were examined through comparison of those with and those without the questionnaire data and through univariate logistic regression with completion of the EQ-5D-3L questionnaire as the outcome measure.

Univariate analysis was conducted through simple linear regression analysis with each self-rated health outcome measure: EQ-5D Index and EQ VAS. For multivariable analysis, first we assessed four aspects of self-rated health determinants: (1) demographic factors, (2) clinical factors, (3) social care factors, and (4) healthcare factors, to identify factors of importance. Subsequently, we fitted the final model using the important variables: UPDRS Parts I-III to represent disease severity due to its clinical importance, and variables with p < 0.1 in the initial aspect-specific multivariable models. For multivariable analyses, the UPDRS parts, age and Zarit carer burden were standardised as z-scores (by mean and standard deviation), and NMSS domain scores converted to percentage of total possible scores to facilitate meaningful coefficients and comparisons. The Schwab and England Scale was not included in multivariable analyses due to overlap with other measures. Variance inflation factors were calculated to check for multicollinearity.

Analyses were conducted as complete case analysis, and a sensitivity analysis was conducted with the same multivariable models run with missing data imputed. Both complete case and imputed datasets have theoretical advantages and disadvantages where data may be missing not-at-random, so the sensitivity analyses quantify these differences in estimated coefficients [34]. Multiple Imputation by Chained Equations was used to impute missing data [35]. This technique was chosen for its flexibility: it can be applied to variables with different characteristics and distributions.

## Results

### Sample

Of 342 participants with PD and cognitive impairment, 277 had been assessed with the EQ-5D-3L questionnaire. Participants were from Germany (n = 78), Portugal (n = 68), Sweden (n = 58), UK (n = 31), France (n = 24) and the Netherlands (n = 18). Of 277 participants, 185 had a pre-established diagnosis of dementia, one had a pre-established diagnosis of Mild Cognitive Impairment (MCI), and 91 scored less than 24/30 on the MMSE in the absence of major depression but without a formal diagnosis of dementia or MCI. Six participants had a disease duration of less than 7 years. 273 had completed all questions of the EQ-5D-3L descriptive system and so have a EQ-5D Index (4 missing); 249 had completed the EQ VAS (28 missing), and both were complete for n = 247. The participants predominantly had severe motor PD as indicated by Hoehn and Yahr staging [36]: n = 28 stages II-III, n = 142 stage IV and n = 107 stage V. The demographic, clinical and resource utilisation findings for the sample (n = 277) are displayed in Table 2, with further detailed breakdowns in Online Resource 4.

**Table 2:** Demographic, Clinical and Care Utilisation Findings

Variable	N (missing)	Mean (sd)	Median (IQR)
**Age** (years)	277 (0)	77.98 (6.94)	78 (74–83)
**Disease duration** (years)	272 (5)	16.32 (7.99)	15 (10–21)
**UPDRS:**			
**Part-I** Mentation, Behaviour and Mood	273 (4)	7.21 (3.03)	7 (5–9)
**Part-II** Activities of Daily Living	266 (11)	29.12 (7.39)	29 (24–34)
**Part-III** Motor Examination	258 (19)	50.84 (15.76)	50 (40–61)
**Part-IV** Complications of Therapy	269 (8)	5.02 (3.39)	4 (2–7)
**Schwab & England**	277 (0)	*(Ordinal)*	30 (20–40)
**Mini Mental State Examination (MMSE)**	255 (22)	18.82 (5.19)	20 (16–22)
**NMSS Total**	229 (48)	126.25 (51.65)	127 (86–161)
**NMSS Domains**			
Cardiovascular	259 (18)	3.88 (5.44)	2 (0–6)
Sleep & Fatigue	257 (20)	14.83 (10.23)	13 (6–21)
Mood & Cognition	261 (16)	23.26 (17.64)	20 (9–34)
Perception	258 (19)	7.62 (8.79)	4 (0–12)
Attention & Memory	258 (19)	20.10 (11.93)	21 (10–30)
Gastrointestinal	260 (17)	12.60 (8.54)	12 (6–18)
Urinary	256 (21)	19.44 (13.28)	18 (8–36)
Sexual Function	244 (33)	11.32 (10.20)	12 (0–24)
Miscellaneous	256 (21)	12.5 (10.09)	12 (4–18)
**Medication (LEDD)**	272 (5)	830.53 (488.72)	752 (495–1051)
**Zarit Burden Score**	200 (77)	33.62 (15.66)	34 (20–46)
**Professional care**^**a**^ (hours per week)	132 (57)	7.77 (22.87)	0 (0–7)
**Informal care**^**a**^ in last 30days (hours)	120 (69)	240.54 (204.14)	180 (81–360)

Variable	Total n (missing)	N (%)	
Healthcare Utilisation for PD in last 3 months			
Inpatient Hospital admission	205 (72)	62 (30.24)	
Primary Care Physician Consultation	199 (78)	113 (56.78)	
Neurologist/Geriatrician Consultation	200 (77)	92 (46.00)	
PD Nurse Consultation	212 (65)	26 (12.26)	
Therapy^b^ Consultation	206 (71)	150 (72.82)	
**Gender** – Male	277 (0)	166 (59.93)	
**Marital Status**	275 (2)		
Single		15 (5.42)	
Married		180 (64.98)	
Divorced		11 (3.97)	
Widowed		62 (22.38)	
Living Apart from Spouse		6 (2.17)	
Living in Stable Partnership without Marriage		1 (0.36)	
**Dementia Medication**^**c**^	273 (4)	115 (42.12)	
**Care Setting**	236 (41)		
Nursing Home		88 (37.29)	
Own Home with Carer		105 (44.49)	
Own Home without Carer		43 (18.22)	
**Carer Relationship**	215 (62)		
No participating informal carer		46 (21.40)	
Spouse or Life Partner		116 (53.95)	
Daughter or Son		44 (20.47)	
Other informal carer		9 (4.19)	

*UPDRS* Unified Parkinson’s Disease Rating Scale; *NMSS* Non-Motor Symptom Scale; *LEDD* Levodopa Equivalent Daily Dose

^a^Applied only to participants living in own home

^b^Includes: Physiotherapy, occupational therapy, speech training, counselling, nursing and massage

^c^Donepezil, rivastigmine or memantine

### Comparison of completers and non-completers

Factors associated with non-completion of the outcome measure (EQ-5D-3L) were worse cognition (MMSE), more severe PD (UPDRS Parts I-III), greater functional dependence (Schwab & England), female gender, nursing home residents, and site differences (higher proportions missing for Nijmegen, Bordeaux and London). Participation of a caregiver, age, marital status, disease duration, non-motor symptoms (NMSS total), and dopaminergic or dementia medication use were not associated with non-completion of the outcome measure.

### Health status outcomes

**Fig. 1:**
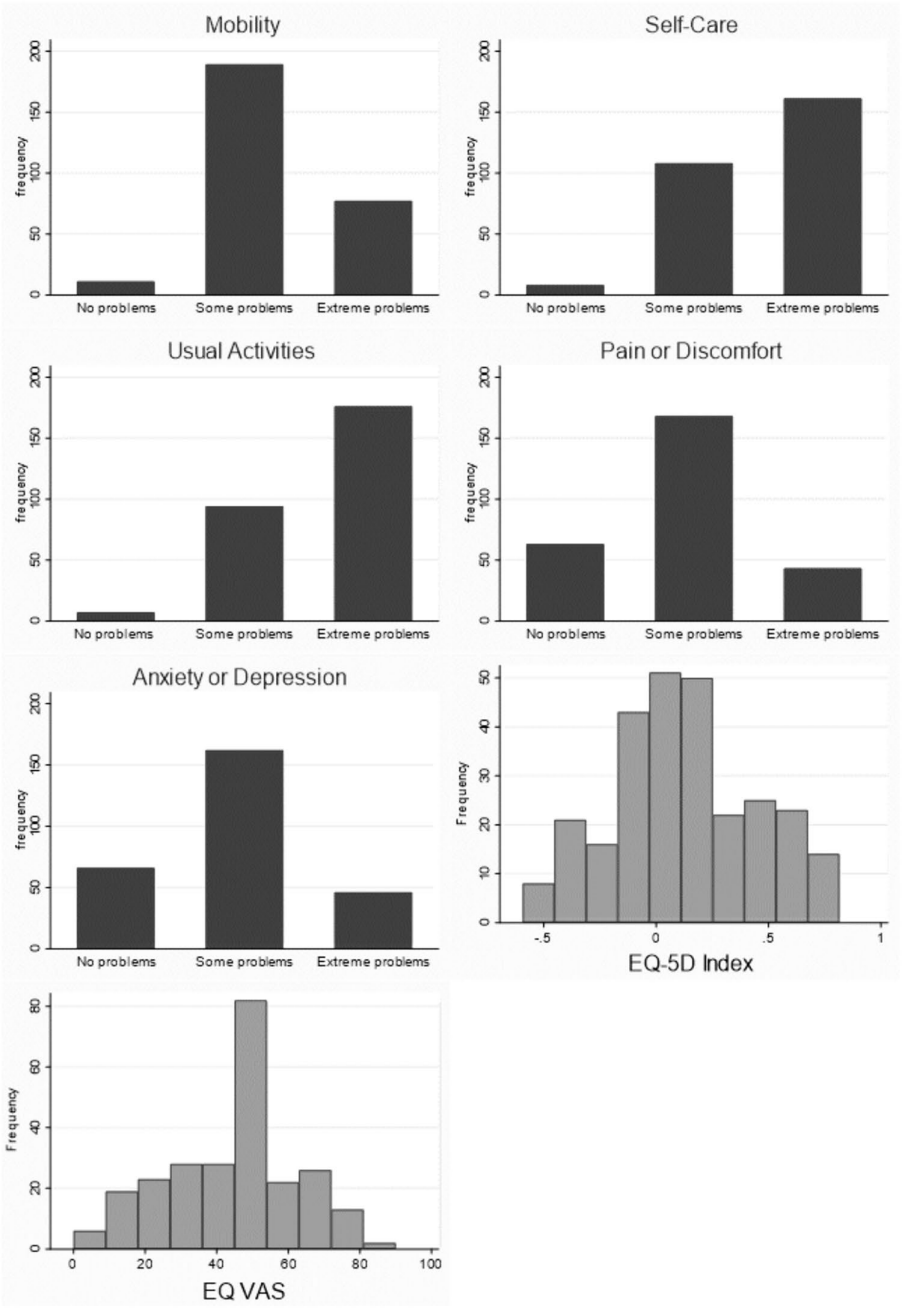
EQ-5D-3L Outcomes: Bar charts of Dimension Response Levels and Histograms of EQ-5D Index and EQ VAS

**Fig. 2:**
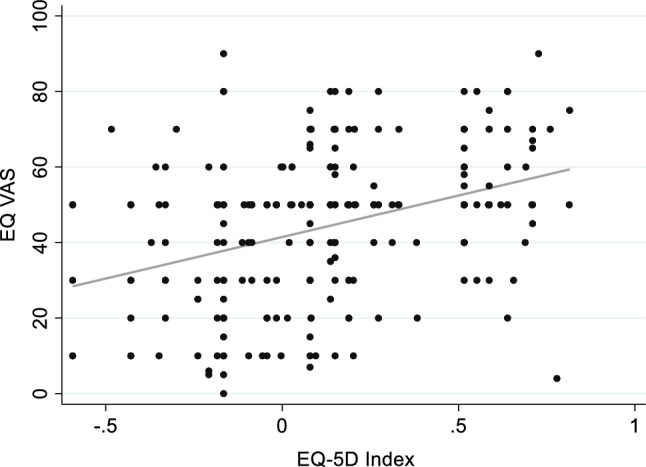
Scatterplot of EQ-5D Index and EQ VAS

Figure 1 displays the self-rated health results for the sample, described by response levels in the five dimensions, the Index (calculated from the dimension scores) and the EQ VAS. Almost all participants (96%) experienced some or severe problems with mobility. Severe problems with self-care and with usual activities were reported by 58% and 64% respectively, and some problems by 39% and 34% respectively. The mean Index was 0.12 (sd 0.33, range -0.59 to 0.82) and median EQ VAS score was 50 (IQR 30–55). Spearman correlation between EQ-5D Index and Visual Analogue Scale showed a moderate positive correlation between the scales, (n = 247, Spearman’s rho 0.3805, P < 0.0001) as illustrated in Fig. 2.

**Fig. 3:**
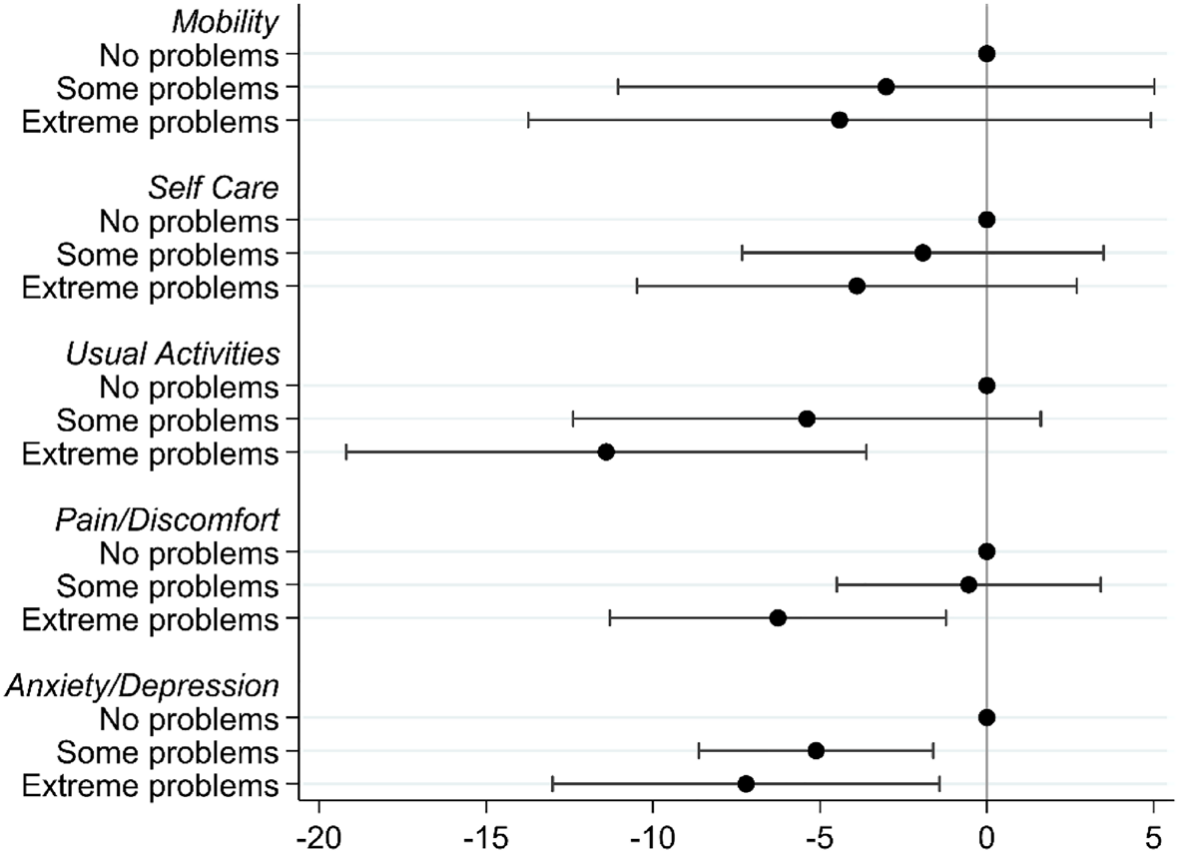
Coefficient plot for regression model showing the relationship between responses on the EQ-5D-3L Descriptive System and the EQ VAS

Analysis of the relative contribution of the five dimensions measured by the EQ-5D-3L descriptive system to the overall self-rated health, measured by the EQ VAS is shown in Fig. 3 (model detailed in Online Resource 1). It demonstrated that being unable to perform usual activities (p = 0.035) and being moderately (p = 0.012) or extremely (p = 0.034) anxious or depressed were significant predictors of the overall self-rated health recorded by EQ VAS.

### Predictors of self-rated health

Univariate analyses are provided in Online Resource 2 and all multivariable analyses are provided in Online Resource 3. On multivariable analysis, no statistically significant associations were identified for demographic or care factors for either outcome measure. For the EQ VAS, non-significant trends toward female gender and greater caregiver burden being associated with worse self-rated health were identified (β = 5.11, p = 0.070 and β = -3.12, p = 0.089 respectively).

Numerous clinical factors were associated with the EQ-5D Index, but few for the EQ VAS. Higher UPDRS Parts-II and -III (ADL and Motor Examination) were significantly associated with worse self-rated health (Index: β = -0.12, p < 0.001 and β = -0.09, p = 0.001 respectively). Mood and perception symptoms were significantly associated with worse self-rated health (β = -0.004, p < 0.001 and β = -0.002, p = 0.04 respectively), whereas gastrointestinal symptoms and higher UPDRS Part-IV (Complications of Therapy) were associated with better self-rated health, for the Index (β = 0.002, p = 0.02 and β = 0.04, p = 0.04 respectively). Only miscellaneous symptoms (which include pain) were associated with worse self-rated health measured by the EQ VAS (β = -0.13, p = 0.05). With regard to healthcare utilisation, having had a PD Nurse consultation in the preceding 3 months was significantly associated with better self-rated health for both outcomes (Index: β = 0.19, p = 0.01; EQ VAS: β = 9.73, p = 0.02).

Important variables from these aspect-specific models were incorporated into a combined model, with UPDRS Parts I-III to control for PD severity, for each outcome, shown in Table 3. Different variables were significant for the different EQ-5D-3L outcomes: UPDRS Part-II (ADL) and -III (motor examination) and NMSS domains mood, perception, and sexual function were all significantly associated with worse self-rated health for the EQ-5D Index; and NMSS gastrointestinal domain and having had a PD nurse consultation in the preceding three months were associated with better self-rated health status for the EQ-5D Index. For the EQ VAS, UPDRS Part-III (motor examination) and NMSS miscellaneous symptom domain were significantly associated with worse self-rated health, whereas higher doses of dopaminergic medication (LEDD) were associated with better self-rated health. The amount of variance accounted for by the combined models (calculated by R^2^) for the EQ-5D Index was 46%, and for the EQ VAS was 24%.

**Table 3:** Multivariable Linear Regression Model (Important Predictor Factors Combined)

Variable	β	95% confidence interval		Standard Error	p
Outcome: EQ-5D Index (n = 172)					
UPDRS Part-I Mentation, Behaviour and Mood (z)	0.03	− 0.03	0.09	0.03	0.33
**UPDRS Part-II Activities of Daily Living (z)**	− 0.10	− 0.16	− 0.03	0.03	**0.003**
**UPDRS Part-III Motor Examination (z)**	− 0.10	− 0.15	− 0.04	0.03	**0.001**
UPDRS Part-IV (z)	0.02	− 0.02	0.06	0.02	0.39
**NMSS Mood domain (%)**	− 0.004	− 0.01	− 0.003	< 0.01	** < 0.001**
**NMSS Perception domain (%)**	− 0.002	− 0.004	− 0.0001	< 0.01	**0.04**
**NMSS GI domain (%)**	0.002	0.001	0.004	< 0.01	**0.01**
**NMSS Sexual function domain (%)**	− 0.001	− 0.002	− 0.0005	< 0.01	**0.003**
**PD Nurse Consultation**^**a**^	0.14	0.01	0.27	0.06	**0.03**
Constant	0.30	0.20	0.40	0.05	0.00
Outcome: EQ Visual Analogue Scale (n = 123)					
Gender: Male	− 0.08	− 6.93	6.78	3.46	0.98
UPDRS Part-I Mentation, Behaviour and Mood (z)	− 4.39	− 9.30	0.52	2.48	0.08
UPDRS Part-II Activities of Daily Living (z)	1.89	− 3.10	6.87	2.51	0.46
**UPDRS Part-III Motor Examination (z)**	− 5.19	− 9.87	− 0.51	2.36	**0.03**
NMSS Urinary domain (%)	0.07	− 0.02	0.16	0.05	0.15
**NMSS Miscellaneous domain (%)**	− 0.19	− 0.36	− 0.02	0.09	**0.03**
Zarit Carer Burden Score (z)	− 3.01	− 6.39	0.36	1.70	0.08
PD Nurse Consultation^a^	7.11	− 2.77	16.99	4.99	0.16
**Parkinson’s Medication (LEDD)**	0.01	0.001	0.02	< 0.01	**0.02**
Constant	40.71	32.17	49.25	4.31	< 0.001

*UPDRS* Unified Parkinson’s Disease Rating Scale; *NMSS* Non-Motor Symptom Scale; *LEDD* Levodopa Equivalent Daily Dose

(z) indicates standardised as z scores

^a^For PD in the preceding three months

### Sensitivity analysis

All models were also run with missing data imputed, detailed in Online Resource 3, with some minor discrepancies identified. The combined models for the EQ-5D Index were similar, but the NMSS perception/hallucinations domain and PD Nurse consultations no longer reached significance with imputed data. The combined models for the EQ VAS were also similar, but the association between UPDRS Part-I and worse self-rated health reached significance with missing data imputed.

## Discussion

### Summary of findings

In patients with cognitive impairment in late-stage PD the health dimensions of Self-Care and Usual Activities as well as Mobility were severely affected, and overall health as rated on the EQ VAS was particularly associated with being unable to perform usual activities and having moderate or extreme anxiety or depression. The two outcome components of the EQ-5D-3L instrument, the Index and EQ VAS score, were associated with slightly different clinical factors. For the Index, both more severe motor and non-motor features (UPDRS Parts-II and -III, NMSS mood, perception and sexual function domains) were associated with worse self-rated health. GI symptoms and having had a recent PD nurse consultation were associated with better self-rated health, although with imputation of missing data the association of self-rated health with PD nurse consultation and with perception symptoms was no longer statistically significant. For the EQ VAS, motor impairment (UPDRS Part-III) and the NMSS miscellaneous symptoms domain, which includes pain, were associated with worse self-rated health. Higher dose of dopaminergic medication was associated with better self-rated health on the EQ VAS, indicating that those who are treated more aggressively experience better health. With missing data imputed, the UPDRS Part-I (Mentation, Behaviour and Mood) was also significantly associated with worse self-rated health on the EQ VAS.

### Context of previous research

Cognitive impairment in PD has been associated with reduced self-rated health in previous studies (e.g. [9, 24, 37]). The descriptive report of self-rated health provided by the EQ-5D-3L in this study allows comparison to other samples, as in Table 4. All dimensions of health showed greater impairment compared to the general European population and to a sample of people with dementia (varied pathologies but predominantly Alzheimer’s disease). Problems with mobility were almost universal, comparable with another PD sample (without dementia), and pain/discomfort scores were also similar [13]. On the other hand, problems with self-care and usual activities were considerably more prominent than in the previous earlier PD sample. There was also an association between not being able to perform usual activities and subjective valuation of overall health status on the EQ VAS, highlighting the importance of independence in daily tasks. In a previous study in PD [24], motor impairment was also an important determinant to self-rated health in patients with PD-Mild Cognitive Impairment but this was not the case in those with PD-dementia. However, motor severity was markedly less in these groups in that study than in our sample. This likely reflects the late-stage of our sample, where motor impairments still play an important role in self-rated health in those with severe cognitive impairment.

**Table 4:** Comparison of EQ-5D-3L Results to the General Population and Other Condition-Specific Samples

	Present study	European general population [66]	PD sample [13]	Dementia sample [67]	Advanced PD sample [68]	Advanced PD sample [69]
**Countries**	UK, Germany, Sweden, the Netherlands, Portugal, France	France, Germany, Italy, Spain, UK	Germany	UK	US, Germany, New Zealand	16 countries^a^
**Key sample information**	n = 277	n = 21,425	n = 75	N = 1547	N = 37	n = 354
	Late stage: 90% Hoehn & Yahr Stage IV-V	Population-weighted	Predominantly mild-moderate disease (Hoehn & Yahr Stage I-III) 68% had MCI, none had dementia	Range of dementia pathologies, but 55% Alzheimer’s disease. Small proportion (2.8%) with PD dementia included	Participants were experiencing motor fluctuations despite optimized medical therapy	Participants were experiencing motor fluctuations despite optimized medical therapy
	Cognitive impairment: MMSE < 24 or diagnosis of MCI or dementia				MMSE < 24 excluded	
**EQ-VAS**	Mean 44.386 (SD 19.393)	Mean 78.3 (SD 20.4)	Mean 65.7 (SD 17.3)	Mean 71.9 (SD 18.6)	Mean 63.9 (SD 14.6)	Mean 50.2 (SD 21.0)
	Median 50 (IQR 30–55)					
EQ-5D-3L Dimension Responses^b^						
**Mobility**	No problems 4%	No problems 87%	No problem 0%	No problems 58%	*Not reported*	
	Problems 96%	Problems 12%	Problems 100%	Problems 42%		
**Self-Care**	No problems 3%	No problems 96%	No problems 72%	No problems 83%		
	Problems 97%	Problems 4%	Problems 28%	Problems 17%		
**Usual Activities**	No problems 3%	No problems 89%	No problems 62%	No problems 66%		
	Problems 98%	Problems 11%	Problems 38%	Problems 34%		
**Pain/Discomfort**	No problems 23%	No problems 72%	No problems 27%	No problems 60%		
	Problems 77%	Problems 28%	Problems 73%	Problems 40%		
**Anxiety/Depression**	No problems 24%	No problems 87%	No problems 72%	No problems 66%		
	Problems 76%	Problems 13%	Problems 28%	Problems 34%		

*MCI* Mild Cognitive impairment; *MMSE* Mini-mental state examination

^a^US, Australia, Canada, Czech Republic, Finland, Germany, Israel, Italy, the Netherlands, New Zealand, Poland, Portugal, Russia, Spain, Thailand, UK

^b^3-Levels dichotomised, collating “some problems” and “severe problems” together to facilitate comparison between studies

Dopaminergic medication, which was associated with better self-rated health, is known to improve motor and some non-motor functions and self-rated health in PD [38], but it becomes more difficult to dose adequately in late-stage PD [2]. Doses typically must be balanced against the risk of adverse effects, including a worsening of neuropsychiatric and cognitive symptoms, often leading to a very cautious approach. Earlier work formed in comparably advanced PD populations revealed that many individuals are relatively undertreated [39]. Our findings are in line with growing recognition that there is still room for therapeutic improvement, even in this complex population with late-stage PD.

Whilst the marked association of depression with poorer self-rated health is consistent with previous reports in both PD [18–20] and in those with cognitive impairment in PD [24], depression/anxiety dimension scores were notably higher than the previous PD sample without dementia. The NMSS mood domain was associated with self-rated health on the EQ-5D Index, and with missing data imputed, ‘Mentation, Behaviour and Mood’ (UPDRS Part-I) was associated with the EQ VAS. This is important as cognitive impairment or dementia can be a barrier to assessment and treatment of depression, although treatment options are available [40]. It highlights that even in the presence of cognitive impairment, depression should be addressed as a treatable symptom.

We did not find significant associations of age and gender with self-rated health on multivariable analyses, although univariate analysis suggested a weak association between male gender and better self-rated health. Disease duration was not significantly associated with self-rated health, unlike in previous studies [8, 24], but all patients were at the late stage of the disease, reflected in the long duration of disease (median 15, IQR 10–21 years).

Perceptual and sexual function symptoms, which we found to be associated with worse self-rated health, were not significantly associated with self-rated health in PD in most previous studies, and where they have, it had been in broader concepts of ‘autonomic symptoms’ [41] and ‘psychiatric symptoms’ [42]. They were not included in the previous studies of self-rated health in PD with cognitive impairment where the importance of these symptoms may be more distinctive. Sexual dysfunction in PD is complex [43, 44]. Although studies are sparse, there seems to be an association between sexual symptoms and depression, anxiety and cognition in PD [45, 46]. A qualitative study in caregivers found that relationship satisfaction and intimacy decreased with evolution of cognitive impairment [47]. Sexual symptoms are under-recognised, under-researched, and undertreated in PD [48], as well as dementia [49], so this finding highlights a need for consideration of sexual symptoms in this population. Psychosis and other neuropsychiatric symptoms are known to be associated with cognitive impairment [50], and have a particular negative impact on caregivers [51].

The association between gastrointestinal symptoms and better self-rated health is surprising. It diverges from previous studies, albeit in different populations [41, 52], although it is interesting to note that this direction of relationship only occurred in our multivariable analysis. In contrast, univariate analysis showed an association between gastrointestinal symptoms and poorer self-rated health, and specifically for constipation, indicating that it may be primarily a function of overall disease severity. This suggests that it is the relative proportion of non-motor symptoms that impact self-rated health, and perhaps gastrointestinal symptoms are relatively well managed amongst these.

Health and social care factors have rarely been reported in past studies, but the importance of the PD nurse seen in our sample was also the case for those with late-stage PD *without dementia* where these factors were included [37]. However, when modelled after multiple imputations, this relationship was not statistically significant, weakening the findings. Since these are both from cross-sectional studies, causation cannot be inferred. Indeed, although there is widespread conviction that PD nurses offer crucial benefits to people living with PD, regardless of disease stage, there is to date only very modest evidence to support this assumption.

### Strengths, limitations and future research

The CLaSP study successfully collected data on a previously understudied population and included a large sample with cognitive impairment, often excluded from research, offering improved generalisability of results to the population of those with late-stage PD and cognitive impairment. Furthermore, our sample had larger numbers with more severe cognitive impairment than previous studies of cognitive impairment in PD (dementia group n = 79 [24] and n = 25 [8]). The use of the EQ-5D-3L instrument facilitates evaluation of important patient-reported outcomes and has been validated in late-stage PD and found to be useful for those with dementia, although is not PD-specific.

Several limitations must be considered. First, this was an observational study so we cannot infer causal relationships between factors. There are likely to be a range of unmeasured factors that contribute further to self-rated health. Of note ethnicity and socioeconomic status data was not collected so could not be incorporated into the models but may play a role in self-rated health, though previous findings reported in the literature are mixed, and these factors are likely less important with increased age [53–55]. Personal factors such as self-efficacy [56], sense of coherence [57] and culture, and from a healthcare perspective, the quality rather than frequency of healthcare contacts may influence self-rated health. The overlap in concepts between the EQ-5D-3L and the clinical measures poses potential risk to the investigation of structural relationships between them, which is a recognised problem, for example, depressive symptoms on the NMSS and the Anxiety & Depression dimension of the EQ-5D-3L. However, PD is a complex condition with a broad spectrum of symptoms, which is why it impacts health so significantly. To exclude all clinical measures that overlap with EQ-5D dimensions from analysis would no longer represent PD. However, our use of initial aspect-specific models helps to disentangle this issue, for example, evaluating the impact of demographic factors independently of clinical symptoms. Moreover, the EQ VAS provides a self-rating of health that is not directly driven by ratings in these dimensions so does not explicitly measure overlapping constructs.

We also recognise limitations of the outcome measure itself: Although well validated, including in related populations [28, 55], the EQ-5D-3L has been seen to have ceiling effects for those with better health, and may overestimate ill health in those with multimorbidity and chronic disease [58]. Furthermore, whilst the use of UK-derived value sets in the calculation of the EQ-5D Index provides consistency, it may not be representative of the values in non-UK countries. Missing data could have introduced information bias, since those with greater severity of PD and cognitive impairment were more likely to have missing outcome data. Some differences were seen on sensitivity analysis, when missing data was imputed, though most findings were consistent with the main analysis.

Future research would benefit from collecting additional data that may influence perception of health, such as ethnicity and self-efficacy; and more information regarding healthcare use, such as quality of consultations and ease of access to services. Intervention studies are warranted, addressing the factors we have identified, to further demonstrate causation, and importantly, to hopefully help improve self-rated health for this late-stage PD population.

## Conclusions

The association between a range of motor and non-motor symptoms and self-rated health affirms the importance for a holistic clinical approach to the management of late-stage patients with cognitive impairment in PD. The relationship between PD nurse consultations and better self-rated health supports the importance of this role but discrepancies on sensitivity analysis warrant further investigation. Investigation of EQ-5D-3L dimensions highlights the widespread and powerful impact on self-care and usual activities, and the importance of usual activities and depression/anxiety to overall self-rated health in people with PD and cognitive impairment. Self-care, usual activities, mood and anxiety should therefore be priorities in clinical practice. A high proportion of variance in self-rated health remains unaccounted for so future research should investigate the role of personal factors and quality of healthcare.

## Supplementary Information

Below is the link to the electronic supplementary material.

Supplementary file1 (PDF 168 KB)

Supplementary file2 (PDF 248 KB)

Supplementary file3 (PDF 365 KB)

Supplementary file4 (PDF 168 KB)

Supplementary file5 (PDF 204 KB)

## Data Availability

Data available upon request.
