# Supplementary material for: Factors associated with self-rated health in people with late-stage parkinson’s and cognitive impairment
Source: Qual Life Res. 2024 Jun 18;33(9):2439–52. doi: 10.1007/s11136-024-03703-2 (PMC11390760; doi:10.1007/s11136-024-03703-2)
Supplement: Supplementary file 1 — Supplementary file1 (PDF 168 KB) [file 11136_2024_3703_MOESM1_ESM.pdf]

Factors Associated with Self-Rated Health in People with Late-Stage Parkinson's and Cognitive Impairment, *Quality of Life Research.* Jennifer S. Pigott, Megan Armstrong, Nathan Davies, Daniel Davis, Bastiaan R. Bloem, Stefan Lorenzl, Wassilios G. Meissner, Per Odin, Joaquim J. Ferreira, Richard Dodel, Anette Schrag.
Correspondence: Prof Anette Schrag, Queen Square Institute of Neurology, University College London, London, UK, a.schrag@ucl.ac.uk

Online Resource 1

Linear regression model investigating association between EQ-5D-3L dimensions and the EQ-VAS

(n=247, R^2= 0.174)

Visual Analogue Scale	β	95% confidence interval		Standard Error	p
EQ-5D-3L Dimension 1: Mobility (reference: no problems)					
Some problems	7.05	-4.43	18.53	5.83	0.23
Confined to bed	2.72	-10.04	15.47	6.47	0.68
EQ-5D-3L Dimension 2: Self Care (reference: no problems)					
Some problems	-3.99	-18.46	10.49	7.35	0.59
Unable to wash & dress	-6.02	-21.25	9.21	7.73	0.44
EQ-5D-3L Dimension 3: Usual Activities (reference: no problems)					
Some problems	-8.90	-24.07	6.27	7.70	0.25
**Unable to perform usual activities**	-16.97	-32.70	-1.25	7.98	**0.04**
EQ-5D-3L Dimension 4: Pain/Discomfort (reference: no pain/discomfort)					
Some moderate pain/discomfort	0.74	-5.16	6.63	2.99	0.81
Extreme pain/discomfort	-6.90	-14.65	0.85	3.93	0.08
EQ-5D-3L Dimension 5: Anxiety/Depression (reference: no anxiety/depression)					
**Moderately anxious/depressed**	-7.33	-13.03	-1.63	2.89	**0.01**
**Extremely anxious/depressed**	-8.38	-16.10	-0.66	3.92	**0.03**
Constant	63.82	43.15	84.49	10.49	<0.001
