# Supplementary material for: Factors associated with self-rated health in people with late-stage parkinson’s and cognitive impairment
Source: Qual Life Res. 2024 Jun 18;33(9):2439–52. doi: 10.1007/s11136-024-03703-2 (PMC11390760; doi:10.1007/s11136-024-03703-2)
Supplement: Supplementary file 2 — Supplementary file2 (PDF 248 KB) [file 11136_2024_3703_MOESM2_ESM.pdf]

Factors Associated with Self-Rated Health in People with Late-Stage Parkinson's and Cognitive Impairment, *Quality of Life Research.* Jennifer S. Pigott, Megan Armstrong, Nathan Davies, Daniel Davis, Bastiaan R. Bloem, Stefan Lorenzl, Wassilios G. Meissner, Per Odin, Joaquim J. Ferreira, Richard Dodel, Anette Schrag. Correspondence: Prof Anette Schrag, Queen Square Institute of Neurology, University College London, London, UK, a.schrag@ucl.ac.uk

Online Resource 2: Univariate Analyses

Univariate Analyses (Simple Linear Regression) with EQ-5D-3L as outcome (both Index and EQ-VAS)

(i) Demographic Variables

	Simple Linear Regression, Outcome: EQ-5D Index			Simple Linear Regression, Outcome: EQ-VAS		
**Variable**	**N (missing)**	**β**	**p**	**N (missing)**	**β**	**p**
**Age** (years) – mean (sd) range	273 (0)	-0.01	0.08	249 (0)	0.17	0.32
**Gender** – n (%)	273 (0)			249 (0)		
Female		(reference)			(reference)	
Male		0.09	**0.03**		1.35	0.59
**Marital Status** – n (%)	272 (1)			249 (0)		
Single		(reference)			(reference)	
Married		0.13	0.13		-0.07	0.99
Divorced		-0.04	0.75		1.14	0.89
Widowed		0.03	0.79		6.21	0.28
Living Apart from Spouse		0.14	0.37		-1.19	0.90
Living in Stable Partnership without Marriage		0.49	0.15		27.14	0.18

(ii) Clinical Variables

	Simple Linear Regression, Outcome: EQ-5D Index			Simple Linear Regression, Outcome: EQ-VAS		
**Variable**	**N (missing)**	**β**	**p**	**N (missing)**	**β**	**p**
**Disease duration** (years) – median (IQR)	268 (5)	-0.00004	0.99	246 (3)	0.32	**0.04**
**UPDRS Part-I** – mean (sd)	271 (2)	-0.04	**<0.001**	247 (2)	-2.13	**<0.001**
**UPDRS Part-II** – mean (sd)	264 (9)	-0.02	**<0.001**	243 (6)	-0.72	**<0.001**
**UPDRS Part-III** – mean (sd)	255 (18)	-0.01	**<0.001**	233 (16)	-0.39	**<0.001**
**UPDRS Part-IV** – median (IQR)	267 (6)	-0.001	0.82	243 (6)	-0.26	0.48
**Schwab & England** – median (IQR)	273 (0)	0.01	**<0.001**	249 (0)	0.44	**<0.001**
**MMSE** – median (IQR)	253 (20)	0.01	**0.004**	234 (15)	0.63	**0.01**
**NMSS Total** – mean (sd)	228 (45)	-0.003	**<0.001**	208 (41)	-0.08	**0.003**
**NMSS Domain Scores:**						
**Cardiovascular** – median (IQR)	257 (16)	-0.003	0.43	236 (13)	0.10	0.68
**Sleep & Fatigue** – median (IQR)	254 (19)	-0.01	**0.001**	233 (16)	-0.33	**0.008**
**Mood & Cognition** – median (IQR)	258 (15)	-0.01	**<0.001**	237 (12)	-0.30	**<0.001**

**Perception** – median (IQR)	255 (18)	-0.01	**0.01**	234 (15)	-0.21	0.15
**Attention & Memory** – median (IQR)	255 (18)	-0.01	**0.001**	235 (14)	-0.38	**0.001**
**Gastrointestinal** – median (IQR)	258 (15)	-0.01	**0.002**	237 (12)	-0.29	0.06
**Urinary** – median (IQR)	254 (19)	-0.01	**0.001**	235 (14)	-0.03	0.73
**Sexual Function** – median (IQR)	242 (31)	-0.01	**0.001**	221 (28)	-0.11	0.39
**Miscellaneous** – median (IQR)	254 (19)	-0.01	**<0.001**	233 (16)	-0.36	**0.004**
**NMSS Individual Question Scores:**						
1. Does the patient experience light-headedness, dizziness, weakness on standing from sitting or lying position?	258 (15)	-0.01	0.26	237 (12)	0.03	0.94
2. Does the patient fall because of fainting or blacking out?	259 (14)	-0.0008	0.92	238 (11)	0.37	0.45
3. Does the patient doze off or fall asleep unintentionally during daytime activities?	260 (13)	-0.02	**0.003**	239 (10)	-0.88	0.005
4. Does fatigue (tiredness) or lack of energy (not slowness) limit the patient's daytime activities?	259 (14)	-0.02	**<0.001**	238 (11)	-0.67	0.02
5. Does the patient have difficulties falling or staying asleep?	260 (13)	-0.0006	0.90	239 (10)	-0.50	0.08
6. Does the patient experience an urge to move the legs or restlessness in legs that improves with movement when he/she is sitting or lying down inactive?	255 (18)	-0.003	0.65	234 (15)	0.03	0.94
7. Has the patient lost interest in his/her surroundings?	260 (13)	-0.03	**<0.001**	239 (10)	-0.79	0.005
8. Has the patient lost interest in doing things or lack motivation to start new activities?	260 (13)	-0.03	**<0.001**	239 (10)	-0.75	0.006
9. Does the patient feel nervous, worried or frightened for no apparent reason?	259 (14)	-0.01	**0.02**	238 (11)	-0.87	0.01
10. Does the patient seem sad or depressed or has he/she reported such feelings?	260 (13)	-0.03	**<0.001**	239 (10)	-1.27	<0.001
11. Does the patient have flat moods without the normal "highs" and " lows"?	259 (14)	-0.02	**<0.001**	238 (11)	-0.95	0.005
12. Does the patient have difficulty in experiencing	259 (14)	-0.02	**<0.001**	238 (11)	-0.64	0.03

pleasure from their usual activities or report that they lack pleasure?						
13. Does the patient indicate that he/she sees things that are not there?	260 (13)	-0.01	**0.01**	239 (10)	-0.55	0.08
14. Does the patient have beliefs that you know are not true?	260 (13)	-0.02	**<0.001**	239 (10)	-0.95	0.005
15. Does the patient experience double vision?	255 (18)	-0.0009	0.87	234 (15)	0.13	0.71
16. Does the patient have problems sustaining concentration during activities?	260 (13)	-0.02	**<0.001**	239 (10)	-0.76	0.01
17. Does the patient forget things that he/she has been told a short time ago or events that happened in the last few days?	259 (14)	-0.01	**0.003**	238 (11)	-0.96	0.001
18. Does the patient forget to do things?	255 (18)	-0.01	0.07	235 (14)	-0.75	0.005
19. Does the patient dribble saliva during the day?	260 (13)	-0.003	0.53	239 (10)	-0.04	0.90
20. Does the patient having difficulty swallowing?	260 (13)	-0.02	**<0.001**	239 (10)	-0.87	0.009
21. Does the patient suffer from constipation?	258 (15)	-0.01	0.05	237 (12)	-0.35	0.23
22. Does the patient have difficulty holding urine? (urgency)	257 (16)	-0.02	**<0.001**	236 (13)	-0.17	0.52
23. Does the patient have to void within 2 hours of last voiding? (frequency)	257 (16)	-0.01	**<0.001**	236 (13)	-0.07	0.78
24. Does the patient have to get up regularly at night to pass urine? (Nocturia)	254 (19)	-0.01	0.17	235 (14)	0.01	0.96
25. Does the patient have altered interest in sex?	247 (26)	-0.01	**<0.001**	226 (23)	-0.36	0.13
26. Does the patient have problems having sex?	242 (31)	-0.02	**<0.001**	221 (28)	-0.05	0.83
27. Does the patient suffer from pain not explained by other known conditions?	259 (14)	-0.02	**<0.001**	238 (11)	-1.13	<0.001
28. Does the patient report a change in ability to taste or smell?	257 (16)	0.0007	0.86	236 (13)	-0.09	0.74
29. Does the patient report a recent change in weight (not related to dieting)?	257 (16)	-0.02	**0.007**	236 (13)	-0.51	0.15
30. Does the patient experience excessive sweating?	259 (14)	-0.01	0.35	238 (11)	-0.50	0.12

(iii) <u>Healthcare Utilisation Variables</u>

	Simple Linear Regression, Outcome: EQ-5D Index			Simple Linear Regression, Outcome: EQ-VAS		
**Variable**	**N (missing)**	**β**	**p**	**N (missing)**	**β**	**p**
**Impatient Hospital admission** – n (%)<br>No<br>Yes	204 (69)	 <br>(reference)<br>-0.03	 <br><br>**<0.001**	182 (67)	 <br>(reference)<br>-4.41	 <br><br>0.16
**Primary Care Physician for PD in last 3months?** – n (%)<br>No<br>Yes	198 (75)	 <br><br>(reference)<br>0.03	 <br><br><br>0.57	178 (71)	 <br><br>(reference)<br>-1.20	 <br><br><br>0.68
**Neurologist/geriatrician for PD in last 3months?** – n (%)<br>No<br>Yes	199 (74)	 <br><br>(reference)<br>0.01	 <br><br><br>0.58	179 (70)	 <br><br>(reference)<br>-3.04	 <br><br><br>0.29
**PD Nurse for PD in last 3months?** – n (%)<br>No<br>Yes	211 (62)	 <br>(reference)<br>0.20	 <br><br>**0.004**	189 (60)	 <br>(reference)<br>8.12	 <br><br>**0.05**
**Therapy[a] for PD in last 3months?** – n (%)<br>No<br>Yes	205 (68)	 <br>(reference)<br>-0.01	0.80	 <br>183<br>(66)	 <br>(reference)<br>-0.66	 <br><br>0.84
**Medication (LEDD)** – median (IQR)	268 (5)	0.00002	0.57	245 (4)	0.004	0.10
**Dementia Medication[b]** – n (%)<br>No<br>Yes	269 (4)	 <br>(reference)<br>0.04	 <br><br>0.36	248 (1)	 <br>(reference)<br>-0.44	 <br><br>0.86

(iv) Social Care Variables

	Simple Linear Regression, Outcome: EQ-5D Index			Simple Linear Regression, Outcome: EQ-VAS		
**Variable**	**N (missing)**	**β**	**p**	**N (missing)**	**β**	**p**
**Care Setting** – n (%)<br>Nursing Home<br>Own Home with Carer<br>Own Home without Carer	234 (40)	 (reference)<br>0.15<br>0.15	 <br>**0.002**<br>**0.02**	211 (38)	 (reference)<br>-1.54<br>4.52	 <br>0.60<br>0.23
**Carer Relationship** – n (%)<br>No participating informal carer<br>Spouse or Life Partner<br>Daughter or Son<br>Other informal carer	213 (60)	 (reference)<br><br>0.08<br>-0.03<br>-0.17	 <br><br>0.16<br>0.66<br>0.16	191 (58)	 (reference)<br><br>-3.65<br>-2.33<br>5.76	 <br><br>0.31<br>0.59<br>0.42
**Zarit Burden Score** – median (IQR)	197 (76)	-0.001	0.44	182 (67)	-0.11	0.23
**Informal care[c]** (	120 (66)	0.0001	0.37	105 (64)	-0.002	0.82
**Professional care[c]** (hours per week) – median (IQR)	131 (55)	-0.0006	0.64	118 (51)	-0.05	0.47

Abbreviations: sd, standard deviation; UPDRS, Unified Parkinson's Disease Rating Scale; NMSS, Non-Motor Symptom Scale; MMSE, Mini-Mental State Examination; LEDD, Levodopa Equivalent Daily Dose. UPDRS parts: Mentation, Behaviour and Mood (Part-I), Activities of Daily Living (Part-II), Motor Examination (Part-III), and Complications of Therapy (Part-IV).

[a]Includes: Physiotherapy, occupational therapy, speech training, counselling, nursing and massage.

[b]Donepezil, rivastigmine or memantine

[c]Applied only to participants living in own home
