# Supplementary material for: Factors associated with self-rated health in people with late-stage parkinson’s and cognitive impairment
Source: Qual Life Res. 2024 Jun 18;33(9):2439–52. doi: 10.1007/s11136-024-03703-2 (PMC11390760; doi:10.1007/s11136-024-03703-2)
Supplement: Supplementary file 3 — Supplementary file3 (PDF 365 KB) [file 11136_2024_3703_MOESM3_ESM.pdf]

Factors Associated with Self-Rated Health in People with Late-Stage Parkinson's and Cognitive Impairment, *Quality of Life Research.* Jennifer S. Pigott, Megan Armstrong, Nathan Davies, Daniel Davis, Bastiaan R. Bloem, Stefan Lorenzl, Wassilios G. Meissner, Per Odin, Joaquim J. Ferreira, Richard Dodel, Anette Schrag. Correspondence: Prof Anette Schrag, Queen Square Institute of Neurology, University College London, London, UK, a.schrag@ucl.ac.uk

Online Resource 3

All multivariable linear regression models with complete case analysis and with missing data imputed.

(i) Model Investigating Demographic Factors - Outcome: EQ-VAS
(ii) Model Investigating Demographic Factors – Outcome: EQ-5D-3L Index
(iii) Model Investigating Clinical Factors - Outcome: EQ-VAS
(iv) Model Investigating Clinical Factors – Outcome: EQ-5D-3L Index
(v) Model Investigating Care Factors - Outcome: EQ-VAS
(vi) Model Investigating Care Factors – Outcome: EQ-5D-3L Index
(vii) Model Investigating Healthcare Factors – Outcome: EQ-VAS
(viii) Model Investigating Healthcare Factors – Outcome: EQ-5D-3L Index
(ix) Combined Model – Outcome: EQ-VAS
(x) Combined Model – Outcome: EQ-5D-3L Index

Notes for all

(z) indicates standardised as z scores

Abbreviations: UPDRS, Unified Parkinson's Disease Rating Scale; NMSS, Non-Motor Symptom Scale; MMSE, Mini Mental State Examination; LEDD, Levodopa Equivalent Daily Dose. UPDRS parts: Mentation, Behaviour and Mood (Part-I), Activities of Daily Living (Part-II), Motor Examination (Part-III), and Complications of Therapy (Part-IV).

(i) Model Investigating Demographic Factors - Outcome: EQ-VAS

Variable	β	95% confidence interval		Standard Error	P
**Complete Case Analysis (n=249, no missing data)**					
Age (z)	0.78	-2.25	3.80	1.54	0.61
Gender: Male	5.11	-0.42	10.64	2.81	0.07
Marital Status (reference: Single)					
Married	-0.71	-11.33	9.90	5.39	0.90
Divorced	2.88	-12.95	18.72	8.04	0.72
Widowed	7.92	-3.81	19.65	5.96	0.19
Living apart from spouse	-1.31	-19.84	17.22	9.41	0.89
Stable Partnership without Marriage	30.28	-9.19	69.74	20.04	0.13
Constant	39.63	28.88	50.39	5.46	<0.001

(ii) Model Investigating Demographic Factors – Outcome: EQ-5D-3L Index

Variable	β	95% confidence interval		Standard Error	P
**Complete Case Analysis (n=272)**					
Age (z)	-0.04	-0.09	0.01	0.03	0.12
Gender: Male	0.06	-0.03	0.14	0.05	0.22
Marital Status (reference: Single)					
Married	0.13	-0.04	0.31	0.09	0.13
Divorced	-0.02	-0.28	0.23	0.13	0.87
Widowed	0.07	-0.12	0.26	0.10	0.47
Living apart from spouse	0.14	-0.17	0.45	0.16	0.38
Stable Partnership without Marriage	0.53	-0.13	1.19	0.34	0.12
Constant	-0.01	-0.18	0.16	0.09	0.92
**Imputed Model (n=273)**					
Age (z)	-0.04	-0.09	0.01	0.03	0.14
Gender: Male	0.06	-0.03	0.15	0.05	0.20
Marital Status (reference: Single)					
Married	0.13	-0.04	0.30	0.09	0.14
Divorced	-0.02	-0.28	0.23	0.13	0.86
Widowed	0.07	-0.12	0.26	0.10	0.48
Living apart from spouse	0.13	-0.17	0.44	0.16	0.39
Stable Partnership without Marriage	0.53	-0.13	1.19	0.34	0.12
Constant	-0.01	-0.18	0.17	0.09	0.94

(iii) Model Investigating Clinical Factors - Outcome: EQ-VAS

Variable	β	95% confidence interval		Standard Error	P
**Complete Case Analysis (n=185)**					
UPDRS Part-I (z)	-2.35	-7.08	2.39	2.40	0.33
UPDRS Part-II (z)	-3.32	-7.83	1.19	2.28	0.15
UPDRS Part-III (z)	-0.76	-4.80	3.28	2.05	0.71
UPDRS Part-IV (z)	-0.41	-3.51	2.68	1.57	0.79
NMSS Cardiovascular domain (%)	0.05	-0.07	0.17	0.06	0.43
NMSS Sleep domain (%)	-0.04	-0.19	0.11	0.07	0.59
NMSS Mood domain (%)	-0.06	-0.19	0.08	0.07	0.43
NMSS Perception domain (%)	-0.02	-0.14	0.11	0.06	0.80
NMSS Memory domain (%)	-0.02	-0.14	0.09	0.06	0.69
NMSS Gastrointestinal domain (%)	0.02	-0.11	0.16	0.07	0.72
NMSS Urinary domain (%)	0.08	-0.004	0.17	0.04	0.06
NMSS Sex domain (%)	0.04	-0.03	0.11	0.03	0.23
**NMSS Miscellaneous domain (%)**	-0.13	-0.26	-0.002	0.07	**0.05**
MMSE	0.20	-0.44	0.85	0.32	0.53
Disease duration	0.15	-0.21	0.51	0.18	0.40
Constant	40.28	24.24	56.31	8.12	<0.001
**Imputed Model (n=249)**					
**UPDRS Part-I (z)**	-3.98	-7.95	-0.02	2.01	**0.05**
UPDRS Part-II (z)	-1.66	-5.60	2.28	2.00	0.41
UPDRS Part-III (z)	-3.20	-6.55	0.16	1.70	0.06
UPDRS Part-IV (z)	-0.53	-3.15	2.10	1.33	0.69
NMSS Cardiovascular domain (%)	0.05	-0.06	0.15	0.05	0.39
NMSS Sleep domain (%)	-0.07	-0.19	0.06	0.06	0.30
NMSS Mood domain (%)	-0.01	-0.14	0.11	0.06	0.83
NMSS Perception domain (%)	0.02	-0.10	0.13	0.06	0.77

NMSS Memory domain (%)	-0.03	-0.13	0.07	0.05	0.56
NMSS Gastrointestinal domain (%)	0.004	-0.11	0.12	0.06	0.94
NMSS Urinary domain (%)	0.06	-0.01	0.13	0.04	0.12
NMSS Sex domain (%)	0.01	-0.05	0.06	0.03	0.86
**NMSS Miscellaneous domain (%)**	-0.13	-0.25	-0.01	0.06	**0.03**
MMSE	0.31	-0.29	0.90	0.30	0.31
**Disease duration**	0.34	0.05	0.63	0.15	**0.02**
Constant	38.96	24.55	53.37	7.30	<0.001

(iv) Model Investigating Clinical Factors – Outcome: EQ-5D-3L Index

Variable	β	95% confidence interval		Standard Error	P
**Complete Case Analysis (n=209)**					
UPDRS Part-I (z)	0.03	-0.04	0.09	0.03	0.40
**UPDRS Part-II (z)**	-0.12	-0.18	-0.06	0.03	**<0.001**
**UPDRS Part-III (z)**	-0.09	-0.14	-0.04	0.03	**0.001**
**UPDRS Part-IV (z)**	0.04	0.001	0.08	0.02	**0.04**
NMSS Cardiovascular domain (%)	-0.0003	-0.002	0.001	0.001	0.67
NMSS Sleep domain (%)	-0.001	-0.003	0.001	0.001	0.50
**NMSS Mood domain (%)**	-0.004	-0.01	-0.002	0.001	**<0.001**
**NMSS Perception domain (%)**	-0.002	-0.003	-0.0001	0.001	**0.04**
NMSS Memory domain (%)	0.001	-0.001	0.003	0.001	0.22
**NMSS Gastrointestinal domain (%)**	0.002	0.0003	0.004	0.001	**0.02**
NMSS Urinary domain (%)	-0.0002	-0.001	0.001	0.001	0.71
NMSS Sexual function domain (%)	-0.001	-0.002	0.00005	0.0004	0.06
NMSS Miscellaneous domain (%)	-0.001	-0.003	0.001	0.001	0.40
MMSE	-0.001	-0.01	0.01	0.004	0.75
Disease duration	0.001	-0.004	0.01	0.002	0.79
Constant	0.33	0.12	0.54	0.11	0.003
**Imputed Model (n=273)**					
UPDRS Part-I (z)	0.003	-0.05	0.06	0.03	0.91
**UPDRS Part-II (z)**	-0.10	-0.16	-0.05	0.03	**<0.001**
**UPDRS Part-III (z)**	-0.10	-0.15	-0.05	0.02	**<0.001**
UPDRS Part-IV (z)	0.02	-0.01	0.06	0.02	0.19
NMSS Cardiovascular domain (%)	-0.0003	-0.002	0.001	0.001	0.75
NMSS Sleep domain (%)	-0.0001	-0.002	0.002	0.001	0.88
**NMSS Mood domain (%)**	-0.003	-0.005	-0.001	0.001	**<0.001**
NMSS Perception domain (%)	-0.001	-0.003	0.0005	0.001	0.18

NMSS Memory domain (%)	0.001	-0.0004	0.002	0.001	0.17
**NMSS Gastrointestinal domain (%)**	0.002	0.00002	0.003	0.001	**0.05**
NMSS Urinary domain (%)	-0.001	-0.002	0.0005	0.001	0.31
**NMSS Sexual function domain (%)**	-0.001	-0.002	-0.0002	0.0004	**0.02**
NMSS Miscellaneous domain (%)	-0.001	-0.003	0.0003	0.001	0.10
MMSE	-0.001	-0.01	0.01	0.004	0.86
Disease duration	0.001	-0.003	0.01	0.002	0.48
Constant	0.30	0.11	0.50	0.10	0.002

(v) Model Investigating Care Factors - Outcome: EQ-VAS

Variable	β	95% confidence interval		Standard Error	P
**Complete Case Analysis (n=128)**					
Caregiver Relationship					
(Reference: No informal carer)					
Spouse of Life Partner	-20.79	-49.19	7.61	-20.79	0.15
Daughter or Son	-22.85	-51.65	5.95	-22.85	0.12
Other informal caregiver	-13.90	-45.48	17.68	-13.90	0.39
Care Setting					
(Reference: Nursing Home or Similar)					
Own home with caregiver	-4.39	-13.10	4.33	4.40	0.32
Own home without caregiver	-1.34	-14.13	11.45	6.46	0.84
Zarit Carer Burden Score	-3.12	-6.73	0.48	1.82	0.09
Constant	69.44	41.97	96.91	13.88	<0.001
**Imputed Model (n=249)**					
Caregiver Relationship					
(Reference: No informal carer)					
Spouse of Life Partner	1.02	-7.35	9.40	4.23	0.81
Daughter or Son	0.56	-8.32	9.44	4.49	0.90
Other informal caregiver	7.72	-7.54	22.98	7.70	0.32
Care Setting					
(Reference: Nursing Home or Similar)					
Own home with caregiver	-0.30	-7.77	7.16	3.76	0.94
Own home without caregiver	5.02	-2.63	12.68	3.88	0.20
Zarit Carer Burden Score (z)	-0.95	-3.80	1.89	1.44	0.51
Constant	42.69	35.78	49.59	3.50	<0.001

(vi) Model Investigating Care Factors – Outcome: EQ-5D-3L Index

Variable	β	95% confidence interval		Standard Error	P
**Complete Case Analysis (n=143)**					
Caregiver Relationship<br>(Reference: No informal carer)<br>Spouse of Life Partner<br>Daughter or Son<br>Other informal caregiver	0.23<br>0.14<br>-0.02	-0.17<br>-0.27<br>-0.48	0.63<br>0.54<br>0.45	0.20<br>0.21<br>0.23	0.26<br>0.51<br>0.94
Care Setting<br>(Reference: Nursing Home or Similar)<br>Own home with caregiver<br>Own home without caregiver	0.02<br>0.01	-0.12<br>-0.20	0.16<br>0.22	0.07<br>0.11	0.75<br>0.94
Zarit Carer Burden Score	-0.04	-0.10	0.02	0.03	0.16
Constant	-0.06	-0.44	0.32	0.19	0.77
**Imputed Model (n=273)**					
Caregiver Relationship<br>(Reference: No informal carer)<br>Spouse of Life Partner<br>Daughter or Son<br>Other informal caregiver	0.08<br>-0.03<br>-0.14	-0.05<br>-0.17<br>-0.38	0.20<br>0.10<br>0.10	0.06<br>0.07<br>0.12	0.23<br>0.64<br>0.27
**Care Setting**<br>(Reference: Nursing Home or Similar)<br>**Own home with caregiver**<br>**Own home without caregiver**	0.12<br>0.18	0.02<br>0.06	0.22<br>0.30	0.05<br>0.06	**0.02**<br>**0.003**
Zarit Carer Burden Score (z)	-0.03	-0.08	0.01	0.02	0.10
Constant	0.02	-0.09	0.12	0.05	0.76

(vii) Model Investigating Healthcare Factors – Outcome: EQ-VAS

Variable	β	95% confidence interval		Standard Error	P
**Complete Case Analysis (n=166)**					
Primary Care Physician consultation[a]	1.27	-4.85	7.40	3.10	0.68
Neurologist/geriatrician consultation[a]	-2.51	-8.35	3.32	2.96	0.40
**PD Nurse consultation**[a]	9.73	1.61	17.84	4.11	**0.02**
Therapy[b] consultation[a]	-0.89	-7.25	5.48	3.22	0.78
Inpatient Hospital Admission[a]	-3.60	-9.93	2.73	3.21	0.26
Parkinson's Medication (LEDD)	0.01	-0.001	0.01	0.003	0.08
Dementia medication[c]	2.20	-3.53	7.94	2.91	0.45
Constant	41.21	33.16	49.25	4.08	<0.001
**Imputed Model (n=249)**					
Primary Care Physician consultation[a]	1.53	-4.39	7.44	2.99	0.61
Neurologist/geriatrician consultation[a]	-1.67	-7.52	4.18	2.96	0.57
PD Nurse consultation[a]	5.87	-2.03	13.78	3.99	0.14
Therapy[b] consultation[a]	0.28	-6.17	6.72	3.25	0.93
Inpatient Hospital Admission[a]	-6.18	-12.45	0.09	3.17	0.05
Parkinson's Medication (LEDD)	0.004	-0.001	0.01	0.003	0.10
Dementia medication[c]	-0.66	-5.60	4.27	2.51	0.79
Constant	41.55	34.06	49.03	3.79	<0.001

[a]for PD in the last 3months

[b]Includes: Physiotherapy, occupational therapy, speech training, counselling, nursing and massage.

[c]Donepezil, rivastigmine or memantine

(viii) Model Investigating Healthcare Factors – Outcome: EQ-5D-3L Index

Variable	β	95% confidence interval		Standard Error	P
**Complete Case Analysis (n=188)**					
Primary Care Physician consultation[a]	0.03	-0.07	0.13	0.05	0.58
Neurologist/geriatrician consultation[a]	0.05	-0.04	0.15	0.05	0.28
**PD Nurse consultation**[a]	0.19	0.05	0.33	0.07	**0.01**
Therapy[b] consultation[a]	-0.03	-0.14	0.07	0.05	0.55
Inpatient Hospital Admission[a]	0.02	-0.08	0.13	0.05	0.65
Parkinson's Medication (LEDD)	0.00003	-0.0001	0.0001	0.00	0.56
Dementia medication[c]	0.07	-0.02	0.17	0.05	0.14
Constant	0.06	-0.07	0.20	0.07	0.34
**Imputed Model (n=273)**					
Primary Care Physician consultation[a]	0.02	-0.07	0.12	0.047	0.62
Neurologist/geriatrician for consultation[a]	0.08	-0.02	0.17	0.049	0.12
**PD Nurse consultation**[a]	0.15	0.02	0.28	0.065	**0.02**
Therapy[b] consultation[a]	-0.02	-0.12	0.08	0.051	0.68
Inpatient Hospital Admission[a]	-0.002	-0.10	0.10	0.051	0.96
Parkinson's Medication (LEDD)	0.00001	-0.0001	0.0001	0.000	0.76
Dementia medication[c]	0.04	-0.04	0.12	0.041	0.36
Constant	0.05	-0.07	0.16	0.058	0.43

[a]for PD in the last 3months

[b]Includes: Physiotherapy, occupational therapy, speech training, counselling, nursing and massage.

[c]Donepezil, rivastigmine or memantine

(ix) Combined Model – Outcome: EQ-VAS

Variable	β	95% confidence interval		Standard Error	P
**Complete Case Analysis (n=123)**					
Gender: Male	-0.08	-6.93	6.78	3.46	0.98
UPDRS Part-I (z)	-4.39	-9.30	0.52	2.48	0.08
UPDRS Part-II (z)	1.89	-3.10	6.87	2.51	0.46
**UPDRS Part-III (z)**	-5.19	-9.87	-0.51	2.36	**0.03**
NMSS Urinary domain (%)	0.07	-0.02	0.16	0.05	0.15
**NMSS Miscellaneous domain (%)**	-0.19	-0.36	-0.02	0.09	**0.03**
Zarit Carer Burden Score (z)	-3.01	-6.39	0.36	1.70	0.08
PD Nurse Consultation for PD in last 3months	7.11	-2.77	16.99	4.99	0.16
**Parkinson's Medication (LEDD)**	0.01	0.001	0.02	0.004	**0.02**
Constant	40.71	32.17	49.25	4.31	<0.001
**Imputed Model (n=249)**					
Gender: Male	-1.01	-5.65	3.63	2.35	0.67
**UPDRS Part-I (z)**	-5.22	-8.11	-2.32	1.47	**<0.001**
UPDRS Part-II (z)	-0.97	-4.35	2.40	1.71	0.57
**UPDRS Part-III (z)**	-4.20	-7.26	-1.13	1.56	**0.01**
NMSS Urinary domain (%)	0.06	-0.01	0.12	0.03	0.09
**NMSS Miscellaneous domain (%)**	-0.18	-0.29	-0.06	0.06	**0.002**
Zarit Carer Burden Score (z)	-0.10	-2.54	2.34	1.23	0.94
PD Nurse Consultation for PD in last 3months	4.07	-2.91	11.04	3.52	0.25
**Parkinson's Medication (LEDD)**	0.01	0.0004	0.01	0.003	**0.03**
Constant	45.05	38.81	51.30	3.17	<0.001

(x) Combined Model – Outcome: EQ-5D-3L Index

Variable	β	95% confidence interval		Standard Error	P
**Complete Case Analysis (n=172)**					
UPDRS Part-I (z)	0.03	-0.03	0.09	0.03	0.33
**UPDRS Part-II (z)**	-0.10	-0.16	-0.03	0.03	**0.003**
**UPDRS Part-III (z)**	-0.10	-0.15	-0.04	0.03	**0.001**
UPDRS Part-IV (z)	0.02	-0.02	0.06	0.02	0.39
**NMSS Mood domain (%)**	-0.004	-0.01	-0.003	0.001	**<0.001**
**NMSS Perception domain (%)**	-0.002	-0.004	-0.0001	0.001	**0.04**
**NMSS Gastrointestinal domain (%)**	0.002	0.001	0.004	0.001	**0.01**
**NMSS Sexual function domain (%)**	-0.001	-0.002	-0.0005	0.0005	**0.003**
**PD Nurse consultation for PD in last 3months**	0.14	0.01	0.27	0.06	**0.03**
Constant	0.30	0.20	0.40	0.05	<0.001
**Imputed Model (n=273)**					
UPDRS Part-I (z)	0.02	-0.03	0.07	0.03	0.55
**UPDRS Part-II (z)**	-0.11	-0.16	-0.05	0.03	**<0.001**
**UPDRS Part-III (z)**	-0.09	-0.14	-0.05	0.02	**<0.001**
UPDRS Part-IV (z)	0.01	-0.02	0.04	0.02	0.62
**NMSS Mood domain (%)**	-0.003	-0.005	-0.002	0.001	**<0.001**
NMSS Perception domain (%)	-0.001	-0.002	0.001	0.001	0.31
**NMSS Gastrointestinal domain (%)**	0.002	0.0001	0.003	0.001	**0.04**
**NMSS Sexual function domain (%)**	-0.001	-0.002	-0.0002	0.0004	**0.01**
PD Nurse consultation for PD in last 3months	0.08	-0.01	0.18	0.05	0.09
Constant	0.28	0.19	0.36	0.04	<0.001
