# Supplementary material for: Factors associated with self-rated health in people with late-stage parkinson’s and cognitive impairment
Source: Qual Life Res. 2024 Jun 18;33(9):2439–52. doi: 10.1007/s11136-024-03703-2 (PMC11390760; doi:10.1007/s11136-024-03703-2)
Supplement: Supplementary file 4 — Supplementary file4 (PDF 168 KB) [file 11136_2024_3703_MOESM4_ESM.pdf]

Factors Associated with Self-Rated Health in People with Late-Stage Parkinson's and Cognitive Impairment, *Quality of Life Research.* Jennifer S. Pigott, Megan Armstrong, Nathan Davies, Daniel Davis, Bastiaan R. Bloem, Stefan Lorenzl, Wassilios G. Meissner, Per Odin, Joaquim J. Ferreira, Richard Dodel, Anette Schrag.
Correspondence: Prof Anette Schrag, Queen Square Institute of Neurology, University College London, London, UK, a.schrag@ucl.ac.uk

Online Resource 4: Demographic & Clinical Details by Stage of Parkinson's & Cognitive Function

	**Age , years** Mean (sd)	**Gender** N, % Male	**Disease duration, years** Mean (sd)	**Dependence, Schwab & England** Median (IQR)
**Whole sample** (n=277)	77.98 (6.94)	166, 60%	16.32 (7.99)	30 (20-40)
**H&Y Stage II** (n=3)	78.33 (5.69)	3, 100%	13.00 (3.00)	40 (40-40)
**H&Y Stage II.5** (n=10)	77.90 (7.92)	7, 70%	12.70 (7.09)	40 (30-40)
**H&Y Stage III** (n=15)	7.420 (8.71)	10, 67%	16.13 (7.98)	40 (40-40)
**H&Y Stage IV** (n=142)	77.71 (6.79)	86, 61%	16.32 (7.59)	35 (30-40)
**H&Y Stage V** (n=107)	78.87 (6.73)	60, 56%	16.79 (8.65)	20 (10-30)
**MMSE >23** (n=48)	76.42 (6.25)	32, 67%	15.61 (7.10)	25 (10-40)
**MMSE 19-23** (n=131)	77.92 (7.48)	79, 60%	15.88 (7.56)	30 (30-40)
**MMSE 14-18** (n=61)	78.10 (6.79)	34, 56%	16.62 (7.79)	30 (20-40)
**MMSE <14** (n=37)	80.03 (5.64)	21, 57%	18.33 (10.50)	20 (20-30)

Abbreviations: H&Y, Hoehn & Yahr Stage; MMSE, Mini Mental State Examination
